# A bioinformatician’s guide to the forefront of suffix array construction algorithms

**DOI:** 10.1093/bib/bbt081

**Published:** 2014-01-10

**Authors:** Anish Man Singh Shrestha, Martin C. Frith, Paul Horton

**Keywords:** suffix array construction, linear-time algorithm, text index, spaced seeds, subset seeds

## Abstract

The suffix array and its variants are text-indexing data structures that have become indispensable in the field of bioinformatics. With the uninitiated in mind, we provide an accessible exposition of the SA-IS algorithm, which is the state of the art in suffix array construction. We also describe DisLex, a technique that allows standard suffix array construction algorithms to create modified suffix arrays designed to enable a simple form of inexact matching needed to support ‘spaced seeds’ and ‘subset seeds’ used in many biological applications.

## INTRODUCTION

The problem of finding the occurrences of a pattern string in a given text is one of the most fundamental computational tasks in bioinformatics. In most bioinformatics applications, the text is a huge database onto which a large volume of pattern queries are thrown. In such cases, precomputing an indexed data structure of the text allows efficient processing of pattern searches.

One simple and effective data structure is a suffix array, which informally is a list of the starting positions of the suffixes of the text, sorted by their alphabetical order. Suffix arrays are easy to understand and implement and form the basis for a host of other sophisticated indexing techniques.

Suffix arrays are related to a slightly more complex data structure known as a suffix tree. Both suffix arrays and suffix trees afford time-efficient solutions to problems of searching for substrings in a text as well as a variety of other related problems. Historically, suffix trees received much attention because time-efficient algorithms for their construction and use were developed early [1]. In bioinformatics, several suffix tree-based applications (e.g. [2]) were developed as well as an influential textbook that largely focused on them [3]. However, suffix trees suffer from a relatively large memory requirement and did not gain widespread popularity. One careful implementation [4] of suffix trees requires 20 bytes per input character in the worst case and in practice, an average of 12.55 bytes per input character for DNA sequences. In contrast, a suffix array in its simplest form only requires 4 bytes per character (for text size 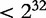). This may not be a fair comparison, as a full-fledged suffix tree is more powerful than a basic suffix array in the sense that it can be used to solve more complex problems. Fortunately, subsequent advances in theory revealed that suffix arrays supplemented with additional tables can substitute for suffix trees [5] and, as we describe here, can be directly constructed in linear time.

The popularity of suffix arrays in bioinformatics is evident from their application in a range of tasks such as pairwise sequence alignment [6–9], error correction of reads from high-throughput sequencers [10, 11], prefix–suffix match finding for genome assembly [12, 13], *k*-mer counting [14] and sequence clustering [15], as well as the development of suffix array software explicitly aimed at bioinformatics applications [16].

A key requirement of any indexing method is that it be constructible in a time- and memory-efficient manner. Progress in the quest for an efficient suffix array construction algorithm started in 1993 with Manber and Myers [17] who applied a prefix doubling technique for repeat detection [18] to suffix array construction, obtaining an 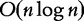 time algorithm for an input text of size *n*. A major breakthrough was achieved a decade later with the almost concurrent discovery of three different linear-time algorithms by Kim *et al.* [19], Kärkkäinen and Sanders [20] and Ko and Aluru [21]. We will not attempt to recount this long history—but instead refer the interested readers to a thorough survey of results up to 2007 by Puglisi *et al.* [22]. Instead, we focus only on linear-time algorithms, and in particular on a recent algorithm called SA-IS proposed by Nong *et al.* [23, 24]. SA-IS, which builds on previous work [21, 25] and their own new ideas, is a beautiful and practical linear-time algorithm. It is among the fastest algorithms available at the time of this writing, and it is also the basis for recent developments in algorithms that simultaneously optimize both time and memory usage [26]. The main goal of this article is to explain SA-IS in a way which can be understood by anyone having a basic background in algorithms. We describe SA-IS in section ‘Suffix array construction’, then discuss and demonstrate the time and memory performance of SA-IS with some simple experiments in section ‘Computational Complexity’.

With biological sequences, the requirement that patterns match ‘exactly’ can sometimes be too strict; rather the search is for regions in the text that approximately match the query. The definition of an approximate match depends on the application at hand, and it determines the feasibility of extending suffix arrays to handle such queries. By a straightforward modification of the lexical ordering of suffixes, suffix arrays can directly support ‘subset’ matching. Subset matching allows matching to ignore differences between some or all characters in a predefined position-specific way. For example, it is possible to construct a modified suffix array that affords efficient search for all suffixes matching (a prefix of) the pattern ‘[ga]..c’, i.e. any occurrence of g or a followed by a c three positions later. Fortunately, as we describe in section ‘Inexact pattern matching’, suffix arrays defined under this kind of modified lexical ordering can be constructed in essentially the same way as conventional suffix arrays [27].

## PRELIMINARIES

Mathematical definitions can be an unpleasant sight; nonetheless, we require a set of definitions and notations that we will use throughout this text. We will present them in this section. We will also use this section to formally introduce suffix arrays and briefly describe their classic application: efficient search of exact matches to substrings in a text.

### Definitions and notations

**Figure 1:**
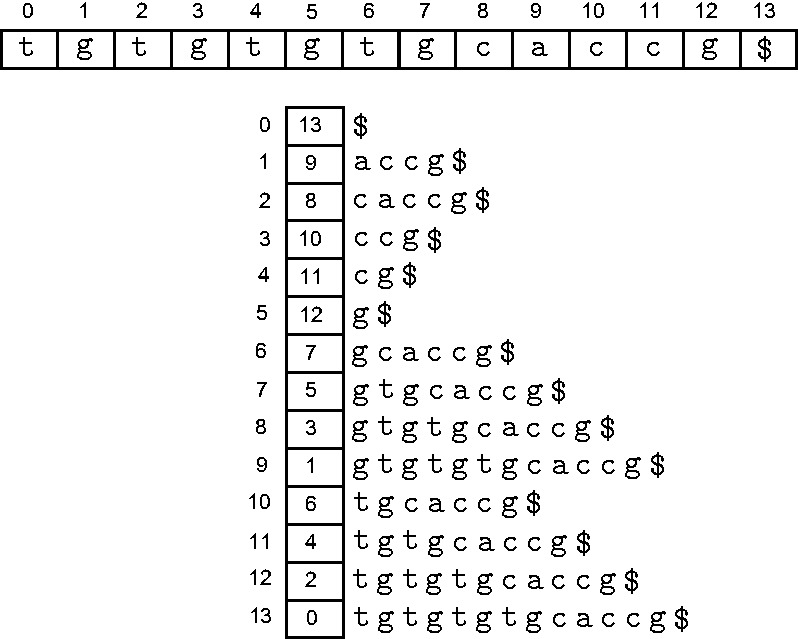
A string (above) and its suffix array (shown vertically) along with the position index on the left and the corresponding suffixes to the right.

Let text *T* be a string of characters 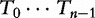, where *T_i_* denotes the *i*th character of *T*. The characters 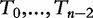 are members of a predefined set of characters called the ‘alphabet’, whereas the end character 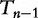 is a ‘sentinel’ character (denoted $) not in the alphabet. For suffix trees, the sentinel is essential for its role in ensuring that no suffix is a prefix of another. They are not absolutely necessary in the discussion of suffix arrays, but are required by some of the construction algorithms. In bioinformatics, the alphabet is usually fixed and relatively small. For example, with DNA strings, the alphabet usually encountered is 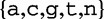, where 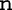 is used at positions where the base has not been confidently identified. The lexical ordering between characters in the alphabet (and therefore for any two strings) is taken to be the same as they would have appeared in a dictionary—except for one extra rule that the sentinel character is defined to be lexically smaller than any other character of the alphabet, or equivalently that if suffix *r* is a proper prefix of suffix *s*, *r* comes before *s* (This is the convention used in the algorithm literature. In practice some software packages adopt the opposite convention, with the sentinel character sorting last.). When applied to strings, we use the symbols <, > and 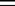 to denote lexical comparison. The ‘size’ or ‘length’ of *T* is the number of characters in *T* and is denoted by 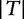. Let 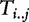 (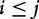) denote the length 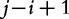 substring of *T* starting at *T_i_* and ending at *T_j_*. Let 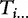 denote the ‘suffix’ 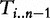 of *T*. The ‘suffix array’ of *T* is the lexically ordered list of its suffixes. Of course, the suffix array does not hold the actual suffixes, but just the index of the starting position of each suffix. An example text with its suffix array is shown in Figure 1.

### Query string search

Given a text *T* along with its suffix array, which we denote here by *SA^T^*, and a query string *P*, we can efficiently locate the occurrences of *P* in *T*. The most straightforward way is binary search. Consider the starting positions of each match of *P* in the text; as *SA^T^* is sorted by suffix lexical order, all suffixes starting with *P* must be in one contiguous block in *SA^T^*. For example, in Figure 1, the starting indices of suffixes with prefix 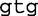 are all clustered within positions 7–9 of the suffix array. Thus, the search for *P* entails finding the two boundaries of this block, i.e. the smallest and largest values of *i* such that *P* is a prefix of the suffix starting at text position 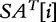. Because the suffixes are in lexically sorted order in *SA^T^*, the two boundaries (or their absence if *P* does not appear anywhere in *T*) can be computed by two rounds of binary searching.

How fast is this search? The size of *SA^T^* is equal to the size of *T*, so a binary search on it requires 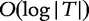 steps. At each step at most 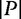 characters need to be compared. Therefore, the time complexity of this search method (excluding the time to enumerate all the occurrences) is 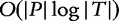. If 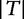 is relatively large, the multiplicative 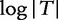 factor might be costly (for example for the human genome, 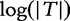 is ∼32).

There are several ways to speed up the search operation, but they come at the cost of memory. A simple method is to cut down on the number of steps required for a binary search by constructing a look-up table that associates a set of *k*-mers with the positions in the suffix array where they first appear as a prefix. Although large values of *k* are prohibitive, this method allows some flexibility to balance the trade-off between search time and memory usage by selecting an appropriate value of *k*.

More sophisticated methods also exist. Manber and Myers [17] show that precomputing the length of the longest common prefix (LCP) for certain pairs of suffixes can reduce the search time to 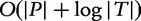. An LCP array stores for each pair of successive suffixes in a suffix array, the length of the LCP between them. Abouelhoda *et al.* [5] show that using an additional table alongside the suffix array and LCP array can bring the time further down to 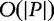, completely removing the dependency on the text size. These methods are attractive because they give meaningful worst case performance guarantees. However, they do require at minimum a few bytes of memory overhead per text character, which can be a practical problem for bioinformatic applications (section ‘Computational Complexity’).

## SUFFIX ARRAY CONSTRUCTION

With a basic understanding of suffix arrays under out belts, we move on to the topic of how to construct them. Given text *T*, a simple way to build its suffix array is to sort the suffixes of *T* using a general string sorting algorithm such as radix sort [28]. This is simple and incurs very little memory overhead for the construction, but its worst case running time is quadratic in the length of the string. Still it is quite fast when the input string does not contain many repeated long substrings. One implementation [6] based on radix sort constructs a suffix array of the human genome in ∼20 min using a decent modern-day computing machine (Intel(R) Core(TM) i7-3770K 3.50 GHz CPU and 32 GB RAM). But note this is for an application in which the n’s do not need to be sorted, otherwise the long runs of nnn 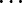 would cause a catastrophic increase in run-time.

Fortunately, the suffixes of *T* are not an arbitrary collection of strings, but rather have the special property of being nested. It turns out that exploiting this property leads to more efficient algorithms, as we describe in this article. In section Bird's-eye view, we briefly outline the first three linear-time algorithms for direct suffix array construction: Kim *et al.* [19], Kärkkäinen and Sanders [20] and Ko and Aluru [21] (Theoretically, linear time can be achieved by first building a suffix tree and traversing it to compute a suffix array. But as suffix trees are memory expensive, this method would largely defeat the whole point of making suffix arrays a practical replacement for suffix trees.). (Linear time can be achieved by first building a suffix tree and traversing it to compute a suffix array, but suffix trees are memory expensive). Then in section ‘A close look at SA-IS’, we give a more detailed description of SA-IS, a recent algorithm proposed by Nong, Zhang and Chan [23, 24]. SA-IS improves on the method of Ko and Aluru, making it one of the fastest algorithms available, not only theoretically but practically as well.

### Bird’s-eye view

Interestingly, around the same time in 2003, three different linear-time methods were proposed independently by Kim *et al.* [19], Kärkkäinen and Sanders [20] and Ko and Aluru [21]. All of them use a similar divide-and-conquer (As the solutions lead to a single-branch recursion, we could use the more precise (but less familiar) term ‘decrease and conquer’) strategy based on the idea that as suffixes are inherently nested, we should be able to determine the lexical order of all suffixes if we knew the order of only a select number of them. The general strategy can be outlined as follows:

Divide phase: Given a text *T* of length *n*, systematically choose a subset 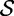 of the suffixes of *T*. Construct a new text 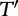 of length 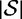 in such a way that sorting the suffixes of 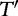 is equivalent to sorting the 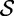 suffixes in the original text *T*.

Conquer phase: Recursively construct the suffix array of 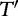. Sorting the suffixes of 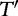 is exactly the same problem (suffix array construction) we started with—albeit under a different alphabet and on a smaller input size.

Combine phase: From the suffix array of 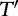, compute the suffix array of *T*.

The algorithms vary in their choices of 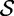, which impacts many things downstream: the construction method and size of 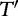, the terminating point of recursion, the complexity of the combine phase and consequently the running time and memory usage. For example, Kim *et al.* [19] take 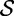 to be the set of even-indexed suffixes, i.e.

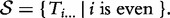

**Figure 2:**
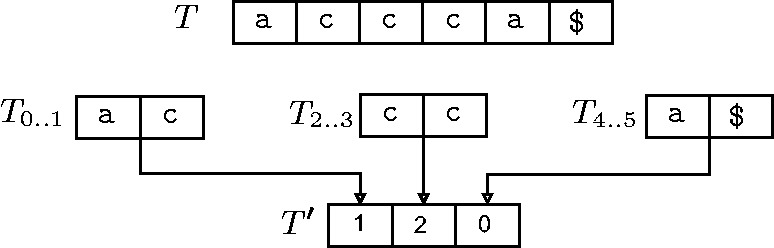
Divide phase of the algorithm by Kim *et al.* Here, 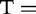 accca$. The set of sampled suffixes 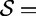
{accca$,cca$,a$}. 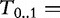 ac; 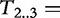 cc; 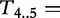
a$. Since a$ < ac < cc, RANK(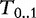)=1, RANK(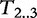)=2, and RANK(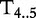)=0. Therefore, 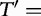 120.

Assuming here for the sake of simplicity that *n* is even, they construct a shorter text 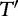 of length 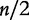 from an alphabet derived from the length two substrings (2-mers) in *T*. More precisely, the *i*th character of 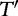 is defined as:

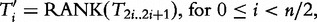

where RANK maps a substring 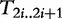 to its rank in the lexical ordering of the set of 2-mers appearing at even index positions in *T*. Figure 2 shows an example of construction of 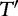 from 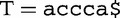.

It is not difficult to see that 
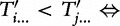
 
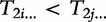
 (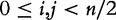), and therefore, we can determine the lexical ordering of 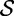 from the lexical ordering of suffixes of 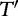. This technique of replacing substrings in an original text by a single character (in a new alphabet) representing the substrings’ lexical order is called ‘lexical naming’, and is a recurring theme in this article. The size of 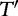 is half that of *T*, thus reducing the problem size by half in each recursion. Unfortunately, the combine phase of the algorithm of Kim *et al.* is extremely complicated.

In contrast, the algorithm by Kärkkäinen *et al.* [20] selects the suffixes as follows:

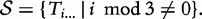

which leads to a simpler divide and combine phase. Although we do not describe their algorithm in detail, we would like to give some intuition for the selection criterion. The key observation is that for any two suffix starting positions 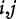: in at least one pair among {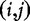, 
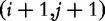
, 
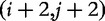
} neither element is an exact multiple of three, and therefore the suffixes corresponding to that pair are both in 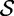. Thus, once the 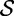 suffixes are sorted, the relative ordering of any two suffixes can be easily determined in constant time. Technically one may say that the set 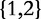 forms a ‘difference cover’ modulo 3, and this strategy can be generalized to covers of modulo larger than three, as described by Burkhardt and Kärkkäinen [29]. Unfortunately, by the construction of Kärkkäinen [20], 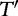 is two thirds the size of *T*, leading to computation time and working memory roughly proportional to 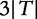. This is not competitive with SA-IS described in the next section, which reduces the problem size to at most one half in each step and is faster and more memory efficient.

### A close look at SA-IS

Following the divide-and-conquer strategy outlined in the previous section, we shall now take a deeper look at the SA-IS algorithm by Nong *et al.* [23, 24]. To maintain a balance between a readable description and a rigorously complete one, we relegate some of the proofs to the Supplementary text.

#### Divide phase

##### Suffix classification and selection

Given a text *T* of length *n*, a suffix 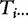 is classified as 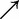 (ascending) type if 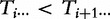 or 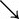 (descending) type if 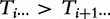. The notation we use here is intended to be graphically mnemonic. Equivalently, the type of 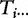 starting with some character, say c, can be defined relative to the next character 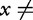 c, following *T_i_* after a run of zero or more c’s. If 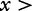 c then 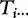 is 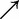, otherwise 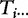 is 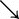. As a special case, the suffix 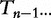 consisting of only the sentinel character is defined to be 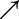. The type of each suffix 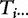 can be computed efficiently by scanning *T* in reverse order and applying the following rule.

**Table:** 

When	classify 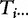 as:
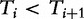	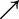 -type
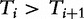	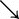 -type
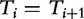	Same as 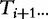

The correctness of the first two conditions is obvious. The correctness of the third condition follows from the observation that if both *T_i_* and 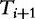 hold the same character, say c, the pair of suffixes 
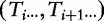
 can be obtained by prepending 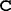 onto the two suffixes 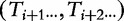.

**Figure 3:**
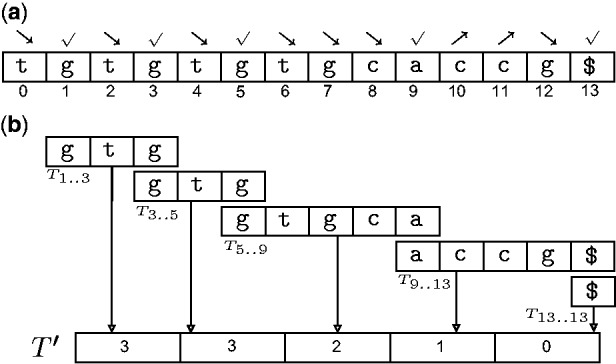
Divide Phase. (a) String *T* with its suffixes classified as 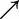, 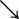, 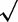. (b) Construction of reduced instance 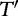 by lexical naming.

A type-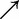 suffix 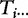 is further classified as a 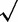 (valley) if 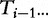 is a 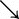 suffix. It might be worth noting that with this definition, 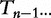 is always a 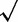 suffix because the sentinel character is always lexically smaller than its preceding character, and on the other hand 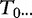, which has no preceding suffix, is not a 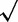 suffix, even when it happens to be an 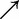 one. From this procedural definition, it is easy to see that we can identify the 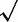-type suffixes by slightly modifying the scan mentioned above. Alternatively, the 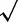 suffix positions can be defined in a more declarative way, as the local minima of the inverse suffix array—the array for which element *i* holds the sorted order rank of suffix 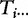. In Figure 3a, we demonstrate the classification of the suffixes of 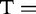 tgtgtgtgcaccg$. We similarly define each character *T_i_* to be of type 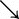 or 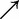 (and possibly also 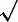) in accordance with the type of suffix 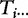. The divide phase of Ko and Aluru’s algorithm selects either the set of 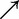-type or 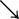-type suffixes (whichever is smaller). Ko and Aluru’s choice results in a simple combine phase (similar to Step 2 of the SA-IS algorithm combine phase described later), but a fairly cumbersome divide phase. SA-IS uses the main idea of the Ko and Aluru combine phase, but selects 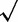-type suffixes instead, and by doing so achieves both simple divide and combine phases.

We close this section by noting a divide-and-conquer strategy does not necessarily imply the use of recursion. Many nonlinear, nonrecursive (or only partially recursive) algorithms also sort a select set of suffices first and then use that information to sort the rest. For example, Itoh and Tanaka [30] select the suffices 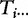 for which 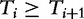, while the algorithms of Mori [31] and Maniscalco and Puglisi [25] use a suffix-selection strategy almost identical to that used by SA-IS.

##### Construction of reduced instance 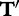


Let us now see how to construct the reduced instance 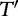 from the 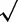 suffixes. Consider the region in *T* starting with a 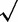 suffix and ending with the next 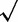 suffix. From the above definitions, it is clear that this region consists of a run of 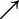 suffixes followed by a run of 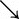 suffixes, and finally a single 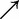 suffix. Again, in the hopes of being graphically mnemonic, we denote the substrings going from one 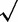 suffix to the next as 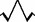 (read ‘w’) substrings. As a special case, 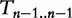 consisting of the sentinel character is also defined to be a 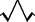 substring.

The 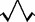 substrings divide *T* into blocks of substrings with overlap of one character (Figure 3b). The 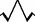 substrings are sorted based on the usual lexical ordering but with one extra rule: if two characters are the same, then we next look at their types, with 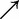 defined to be larger than 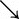. For example in Figure 3, 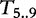 sorts before 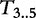 as *T*_7_ is 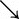 while *T*_5_ is 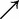. These rules allow us to sort the set of 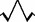 substrings in *T* and from that obtain lexical names for each 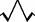 substring. 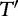 is obtained by concatenating the lexical names of the 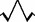 substrings in the order they appear in *T*. (Figure 3b).

The innocent-looking ‘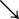 sorts before 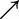
’ rule is in fact important. The intuition behind it is that between a pair of 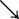-type and 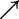-type suffixes of *T*, both starting with the same character, the 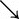 suffix is lexically smaller than the 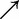 one (Lemma S1). Thus, the lexical order of two suffixes of *T* will be correctly reflected in the order of their corresponding suffixes in 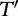. We provide a formal proof of this in the Supplementary text (Theorem S1). What is perhaps more subtle is that this extra rule eliminates the proper prefix problem that is inherent with lexical naming of variable-length substrings, by telling us if the prefix should come before or after the substring which contains it (section 'The Proper prefix problem' in supplementary material gives an example and more formal discussion of this observation).

At this point, there is one major outstanding issue. For this algorithm to achieve a linear run-time, we must be able to sort the 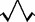 substrings in linear time. While it is easy to sort the 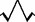 substrings in quadratic time, it is not straightforward how to accomplish this in linear time. Nong *et al.* found a surprisingly simple solution, which is nearly the same as the combine phase described in section ‘Combine phase’. For completeness, however, we explicitly describe the linear-time sorting of the 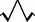 substrings in the Supplementary text (Section S1.3).

#### Conquer phase

If there are no ties in the sorting of the 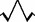 substrings (in other words each lexical name is unique), the order of the 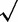 suffixes can be determined without the need for further recursion. Otherwise, the suffix array of 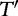 is computed recursively.

#### Combine phase

The recursion returns the order of the suffixes of 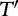, which tells us the relative order of the 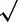-type suffixes of *T*. We wish to use this information to order all the suffixes of *T*.

**Figure 4:**
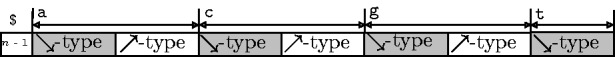
Buckets of a DNA-string suffix array of length *n*. Gray indicates 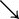-type positions. The bucket for *T* does not have a subbucket for 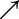 because there cannot be any 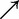 suffix starting with the lexically greatest character of the alphabet.

Even without this new information, we can say a few things about the suffix array of *T*. First, all suffixes starting with a given character will be in a contiguous block. Second, as was mentioned earlier, between a pair of 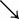-type and 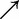-type suffixes, both starting with the same character, the 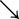 suffix is lexically smaller than the 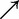 one (Lemma S1). Therefore, the suffix array of *T* can be thought of as being partitioned into buckets, every bucket holding all the suffixes starting with the same character; and each bucket further partitioned into two sub-buckets, one for the 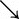-type which is to the left of the one for the 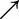-type suffixes. For example, if *T* is a DNA string, its suffix array can be logically partitioned (with some buckets possibly empty) as shown in Figure 4.

To construct the suffix array of *T*, we start by allocating an array *A* the size of *T*. *A* will eventually end up as the suffix array. The procedure can be explained in three major steps described below. A running example with the text *T* from Figure 3 is provided in Figures 6 and 7. In the following, we will refer to the character in the *i*th position of *A* as *A_i_*.

**Figure 5:**
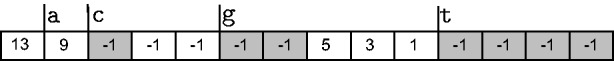
Array *A* at the end of Step 0 in which the 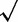 suffixes have been placed in their buckets in sorted order. Gray indicates 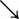-type positions. This order of 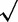 suffixes is obtained from recursion.

**Figure 6:**
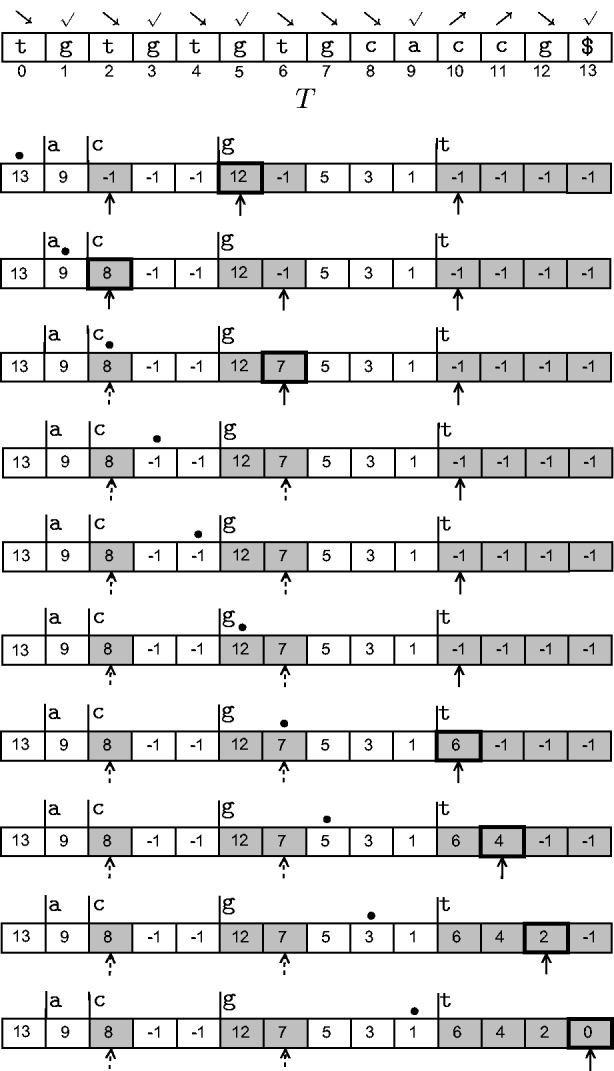
Animation of Step 1 of the combine phase as the sweep proceeds from left to right. The original text *T* is also shown for reference. The 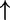 symbols point to the current heads of 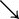-type subbuckets, the ∙ symbol shows the current position of the sweep and cells with thick boundaries indicate changes. For example, in the topmost row, suffix index 13 is encountered; and as 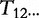 is 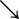-type, 12 is inserted at *A*_5_, the current head of the bucket for 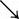-type suffixes starting with g. The sweep proceeds accordingly. Whenever a pointer reaches the edge of its bucket, we change its representation to a dashed arrow. From sweep position 10 onwards, the array does not change and so this animation excludes those steps.

**Figure 7:**
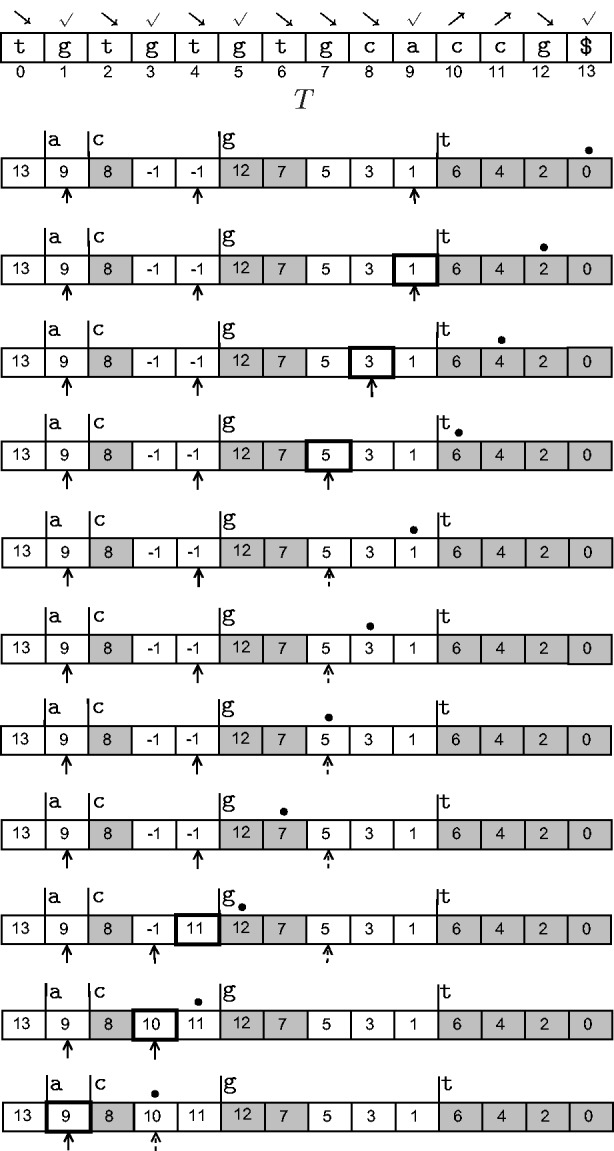
Animation of Step 2 of the combine phase as the sweep proceeds from right to left. The original text *T* is also shown for reference. The 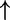 symbols point to the current tails of 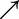-type subbuckets, the ∙ symbol shows the current position of the sweep, and cells with thick boundaries indicate changes. For example, in the topmost row, suffix index 0 is encountered, and therefore no action needs to be taken. Next, suffix index 2 is encountered; and as 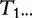 is 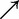-type, 1 is inserted at *A*_9_, the current tail of the bucket for 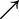-type suffixes starting with g. Whenever a pointer reaches the edge of its bucket, we change its representation to a dashed arrow. From sweep position 2 onwards, the array does not change and so this animation excludes those steps.

**Step 0:** This step initializes *A*. Set all elements of *A* to the special value of −1. Compute the bucket boundaries of *A*, by counting the frequency of each character type pair (e.g. a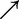) in the text. Pointers can be used to mark the boundaries—one for each 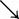-type bucket pointing to its left end (head). (After Step 1 below, these pointers can be reused to point to the right end (tail) of each 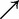-type bucket). Place the 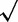-type suffixes into the ends of their buckets in their sorted order (Figure 5). Note that this is not the final resting position of the 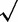-type suffixes.
**Step 1:** This step uses the order of 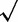-type suffixes to sort the 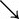-type suffixes. Scan *A* from left to right, skipping any elements with value −1. For each suffix index *A_i_* encountered, if 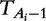 is 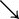, place 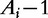 at the current head of its respective bucket, and then increment that head pointer (Figure 6).
**Step 2:** This step uses the order of the 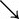-type suffixes obtained from Step 1 to sort the 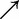-type suffixes. Scan *A* from right to left. For each suffix index *A_i_* encountered, if 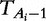 is 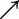-type, place 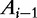 into the current tail of its respective bucket and decrement that tail pointer (Figure 7).

At the end of Step 2, *A* is exactly the suffix array of *T*!

Although these steps consist of simple operations, the correctness of Steps 1 and 2 might not be immediately clear. Informally, Step 1 is a clever one-sweep implementation of the following idea. The order of two 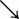-type suffixes 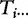 and 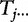, starting with the same initial character, can be determined recursively as 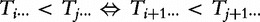. We can end this recursion when we reach the smallest integer *x* such that 1) 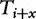 and 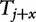 are different characters or 2) at least one of them is of 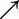-type (i.e. starts a 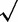-type suffix). If exactly one is of 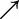-type suffix, it follows immediately that it goes after the other in the suffix array, while if both of them start a 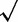-type suffix, their order is already known from the conquer phase. Step 2 can be understood similarly—it sorts the 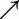-type suffixes inductively based on the order of 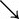-type suffixes. A formal proof of correctness of the combine phase is provided in the Supplementary material (Theorems S3 and S4).

In fact, SA-IS uses an almost-identical ‘induced-sorting’ procedure to sort the 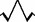 substrings in the divide phase as well (section ‘The Method’ in supplementary material). Finally, we know what the ‘IS’ in SA-IS stands for!

## Computational Complexity

### Time complexity

The time complexity of SA-IS is linear in the input text size. This is because each divide phase results in the problem being reduced into a problem of size half or even smaller (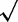 suffixes occur at boundaries between 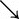 and 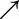 suffixes and therefore at most ½ of all suffixes can be 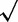 suffixes), and the additional work of dividing and combining at each level can be performed in linear time.

### Memory usage

The suffix array of a length-*n* text can be stored in 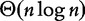 bits of space—the suffix array holds *n* numbers, and each number can be encoded using 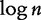 bits. Because the suffix array itself does not contain the text itself, we also need to load the text into main memory to be able to process queries. This requires another 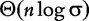 space, where σ is the size of the alphabet.

In many bioinformatics applications, the text length is shorter than 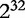, allowing each index to be represented using 4 bytes. Also, the alphabet size is small enough that 1 byte is enough to represent each character. This adds up to a total of 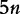 bytes. A haploid human genome contains ∼3 billion bases, and therefore it requires a total of 15 GB of memory just to hold the text and suffix array. Moreover some genomes are 10s or even 100s of times larger than that of human, and of course one may want to index multiple genomes. Thus minimizing memory use is an important concern when using suffix arrays.

There are several possible workarounds, sometimes at the cost of higher query processing times. In many cases, the data can be partitioned into logical segments (e.g. chromosomes in a genome), and the index for each partition can be treated separately. In applications where some loss of information is tolerable, one can choose to store only a subset of the suffixes (e.g. only every second suffix), an idea known as a sparse suffix array [32]. Sparse indexing is not unique to suffix arrays, and in fact is used by several sequence alignment tools, e.g. BLAT [33], indexed MegaBLAST [34]. More sophisticated, suffix-array-like, reduced memory data structures have been developed, including methods involving the Burrows–Wheeler Transform [35], FM-index [36] and compressed suffix arrays [37]. These methods typically reduce memory use at the expense of the computation time needed for pattern searches. Vyverman *et al.* [38] give a comprehensive review of the trade-offs offered by these and other indexes.

Apart from the storage memory of the index itself, we also need to consider the working memory required by the construction algorithm. This is defined as the additional memory required by the construction algorithm, excluding the memory used to hold the input text and the output suffix array. Various ‘lightweight’ suffix array construction algorithms have been proposed (e.g. [39, 40]) which achieve reduced working memory.

The SA-IS algorithm is elegant not only in terms of computation time, but also in the way it leads to an implementation with most of the working memory allocated to bucket pointers. Assuming the original text *T* is from a small fixed alphabet, the number of distinct characters in the reduced text 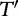 can become as large as 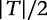, and therefore, the bucket pointers for the first level of recursion can require almost 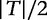 buckets (often considerably less in practice), leading to a working memory of roughly 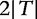 bytes (with 4-byte pointers). Surprisingly, no working memory is needed to hold the text or suffix arrays computed during recursion, as these can be computed using the same memory that ultimately holds *SA^T^*. The fact that this can be done is not obvious and somewhat involved, so we provide a detailed analysis of the memory usage of SA-IS in the Supplemental material ‘Memory usage of SA-IS’. Intriguingly, Ge Nong has recently reported a new suffix array construction algorithm, SACA-K [26], which achieves an *O*(1) working space for constant alphabet, while maintaining linear runtime. SACA-K can be understood as a variant of SA-IS, modified with clever optimizations to eliminate the need for separate memory for bucket pointers after the first level of recursion. A full description is beyond the scope of this article, but a thorough reading of this review should be of great help in understanding SACA-K.

### Benchmarking

To understand the time and memory performance of SA-IS in practice, we performed simple experiments with several biological data sets that are representative of the kind of data usually encountered in bioinformatics research. For more comprehensive benchmarks on general applications, we refer the reader to [22, 24].

#### Data sets

The data sets used are summarized in Table 1. The last entry in the table requires some explanation. We started with chr22 of the human genome. Using Dnemulator [41], a package for simulating polymorphisms, we simulated several copies of chr22, as if they were coming from different individuals. Dnemulator does this by picking real alleles based on their frequencies as reported in snp132Common.txt, a SNP database [42] available from the UCSC Genome Database. In this manner, we constructed four different data sets containing 1, 3, 5 and 7 different copies of chr22. This data set is relevant to bioinformatics because with increasing amount of sequence data becoming available, it is likely that data sets contain genomic sequences from different individuals of the same species and/or from similar organisms. Another motivation for this experiment is to illustrate the problem a nonlinear, but usually fast, suffix array construction algorithm exhibits when faced with many suffix pairs that share long common prefixes. Many biological sequences such as genomic sequences contain many long repeats and are especially prone to long common prefixes. For algorithms that directly compare suffixes to sort them, this means the comparison takes longer.

**Table 1:** Different biological data sets used for tests

Data set	Size: (roughly 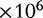 characters)	Obtained from
*D. melanogaster* (fruitfly) genome	165	ftp://ftp.ensembl.org/pub/release-73/fasta/drosophila_melanogaster/dna/
*G. gallus* (chicken) genome	992	ftp://ftp.ensembl.org/pub/release-73/fasta/gallus_gallus/dna/
UniProtKB/Swiss-Prot protein data set	193	http://www.uniprot.org/downloads
UniProt fungi proteins data set	872	http://www.uniprot.org/uniprot/?query=taxonomy%3a4751&format=*
Human chromosome 22 and its copies	36 to 249	http://hgdownload.cse.ucsc.edu/goldenPath/hg19/bigZips/ and simulation (see text)

#### Preprocessing the data sets

For DNA data, usually the character 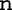 appears wherever the nucleotide at that position has not been correctly identified. We removed all occurrences of 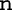 and reformatted the data files as suitable for each software package to represent the biological sequences as a single concatenated text string with a unique delimiter character placed between adjacent sequences.

#### Programs

We benchmarked several freely available suffix array construction programs based on different algorithms, including two implementations of SA-IS: one available from the authors of SA-IS and an implementation by Yuta Mori (https://sites.google.com/site/yuta256/sais). We also tested SACA-K [26], a recently published memory-efficient successor of SA-IS and included an implementation of the Deep-Shallow algorithm [40], which is theoretically not a linear-time algorithm, but has been shown to be fast and lightweight in practice [22]. For each program, we used the default parameter settings. Finally, for baseline comparison, we included our implementation of radix sort. The links to the programs tested here are available in the Supplementary material.

#### Results

**Figure 8:**
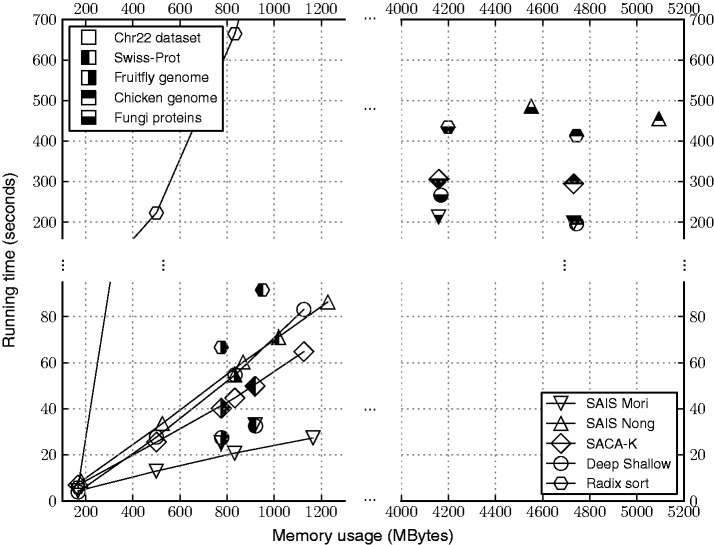
Time and memory performance of implementations of select suffix sorting algorithms. The shapes of the markers distinguish the different programs, and the fill-styles distinguish the data sets. The nonfilled markers connected by lines correspond to the performance for the four data sets constructed from increasingly many polymorphic copies of human chromosome 22.

We ran the programs on a machine Intel(R) Core(TM) i7-3770K 3.50 GHz CPU and 32 GB RAM with a linux-based OS installed. We measured running time using the 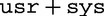 time reported by the Linux 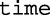 command, and peak memory usage using the Linux 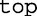 command. The running time and peak total memory usage of each program for each data set is shown in Figure 8.

The SA-IS implementation by Mori shows superior time and memory performance across all data sets. However, when compared across implementations from the same group (Nong *et al.*), SACA-K outperforms SA-IS in both time and memory. Compared with Mori’s implementation of SA-IS, Deep Shallow performs significantly poorly for the chr22 data set, but it is fairly competitive for the other data sets. Radix sort is slow for the chr22 data set, as one might expect. For the remainder of the data sets, it is not far off from the other methods. However, we should note that the implementation of radix sort we used has an application specific advantage in that it does not fully sort the suffixes, but instead only sorts up to the first delimiter (i.e. does not sort past the boundaries of the biological sequences forming the input text).

## INEXACT PATTERN MATCHING

With biological sequences, we are not always looking for exact matches. As a simple example, consider searching for a given string in a protein-coding DNA sequence. Protein-coding DNA tends to exhibit substitution at the third position of every codon, as this often does not affect the encoded amino acid. We could therefore relax our pattern-matching requirements by allowing a mismatch at every third position of the pattern, for example with a text 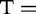 actcgtact, the substring 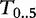 would be a match for the query pattern 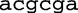.

Approximate matching comes in different flavors, necessitating appropriate modification to ordinary suffix arrays. Here we deal with three kinds of approximations: spaced seeds that allow any mismatches at predetermined positions, subset seeds that allow only certain kinds of mismatches at predetermined positions and finally matches that are within a prescribed edit/Hamming distance.

### Spaced seeds: patterns with don’t-care positions

The concept of ‘spaced seeds’ is widely used in pairwise sequence alignment algorithms that use BLAST-like seed-and-extend techniques. Given two sequences *T*_1_ and *T*_2_, these alignment algorithms first identify potentially similar regions using ‘seeds’, short strings that can be found in both *T*_1_ and *T*_2_. Originally seeds were required to match exactly, but it has since been shown that the sensitivity of these alignment algorithms increases significantly when spaced seeds that allow mismatches at certain positions are used [43–45]. This has resulted in a host of sequence alignment tools that rely on the concept of spaced seeds (for example, see [6, 44, 46, 47]). Spaced seeds have also been applied to the problem of correcting errors in reads from high-throughput sequencers [48].

Given the tremendous interest in spaced seeds, it is desirable to have a suffix array-like data structure that when constructed for a database string and a set of don’t-care positions facilitates rapid pattern searches. We devote the rest of this section to describing the construction of such an index called the ‘spaced suffix array’. First, let us start with some mathematical definitions.

#### Definitions…again

The don’t-care positions of a spaced seed can be described by a ‘mask’ *M*, a binary vector represented by a string over the alphabet 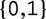, with the 0 positions of *M* corresponding to the don’t-care positions. Applying *M* to a length-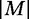 substring 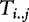 of a string *T* results in a ‘masked substring’ 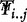, which is the string obtained from 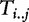 by replacing 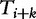 for each 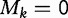 (i.e. the characters corresponding to the don’t-care positions) by a unique fixed character. As an example, let 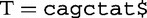, 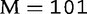 and * be the replacement character, then applying *M* to a substring of *T*, say 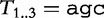, we get the masked substring 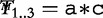.

The definition of masked substring can be extended to substrings that are not of length 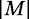 in the following manner. If 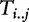 is shorter than *M*, we apply only the prefix of *M* which has the same length as 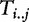. If 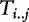 is longer than *M*, we apply the mask cyclically as many times as required, so for example the mask 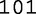 can be thought of as 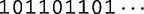. Using this mask and the same *T* as before, 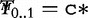 and 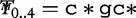.

**Figure 9:**
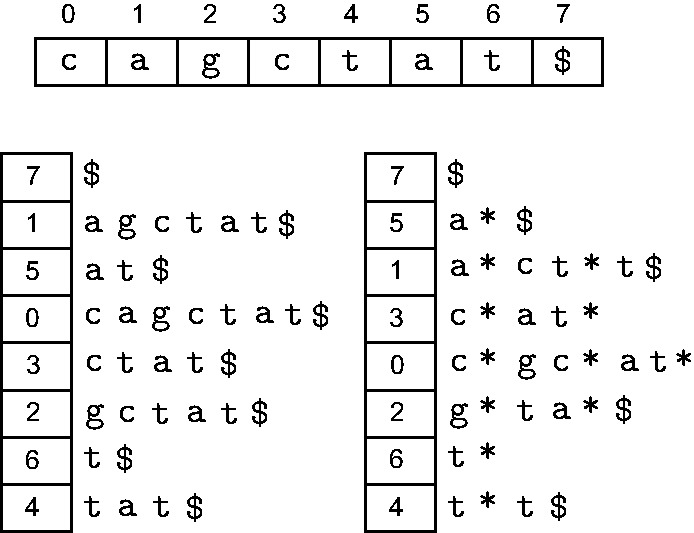
Contrasting the ordinary suffix array (left) of cagctat$ with its spaced suffix array under mask 101 (right). The characters at don’t-care positions have been replaced by *.

This definition naturally extends to the concept of a ‘masked suffix’ of a suffix 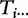, which we shall denote by 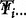. The ‘spaced suffix array’ of *T* under mask *M* is a list of the masked suffixes of *T* sorted in their increasing lexical ordering. Depending on the mask and text, a spaced suffix array is in general different from an ordinary suffix array of the same text. Figure 9 shows one such example with 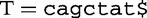 and 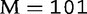.

#### Query processing in a spaced suffix array

Because a spaced suffix array is constructed with a predetermined don’t-care pattern in mind, naturally the query string must also be processed under the same pattern. Apart from this, processing queries in a spaced suffix array is similar to what is done with ordinary suffix arrays, a topic we discussed in section ‘Query string search’.

#### Constructing a spaced suffix array

Given a text *T* and a mask *M*, a straightforward solution to computing the spaced suffix array of *T* under mask *M* is to use radix sort that skips the don’t-care positions. As we have seen in section ‘Benchmarking’, radix sort can become slow for certain inputs, and therefore faster solutions are desirable. Horton *et al.* [27] describe a method called ‘DisLex’, which uses lexical naming to transform the input text into a new ‘DisLex text’ (over a new alphabet), such that the desired spaced suffix array can be easily derived from the ‘ordinary’ suffix array of the DisLex text. This method constructs a masked suffix array in three steps:

**Step 1:** Transform *T* to a new text 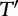 of similar length such that sorting the masked suffixes of *T* is equivalent to sorting the ordinary suffixes of 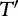. The core idea uses lexical naming, a technique we came across while discussing suffix array construction algorithms (e.g. Figures 2 and 3).
**Step 2:** Apply any linear-time suffix array construction algorithm on 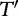.
**Step 3:** Reverse-transform the suffix array of 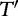 to obtain the spaced suffix array of *T*.

**Figure 10:**
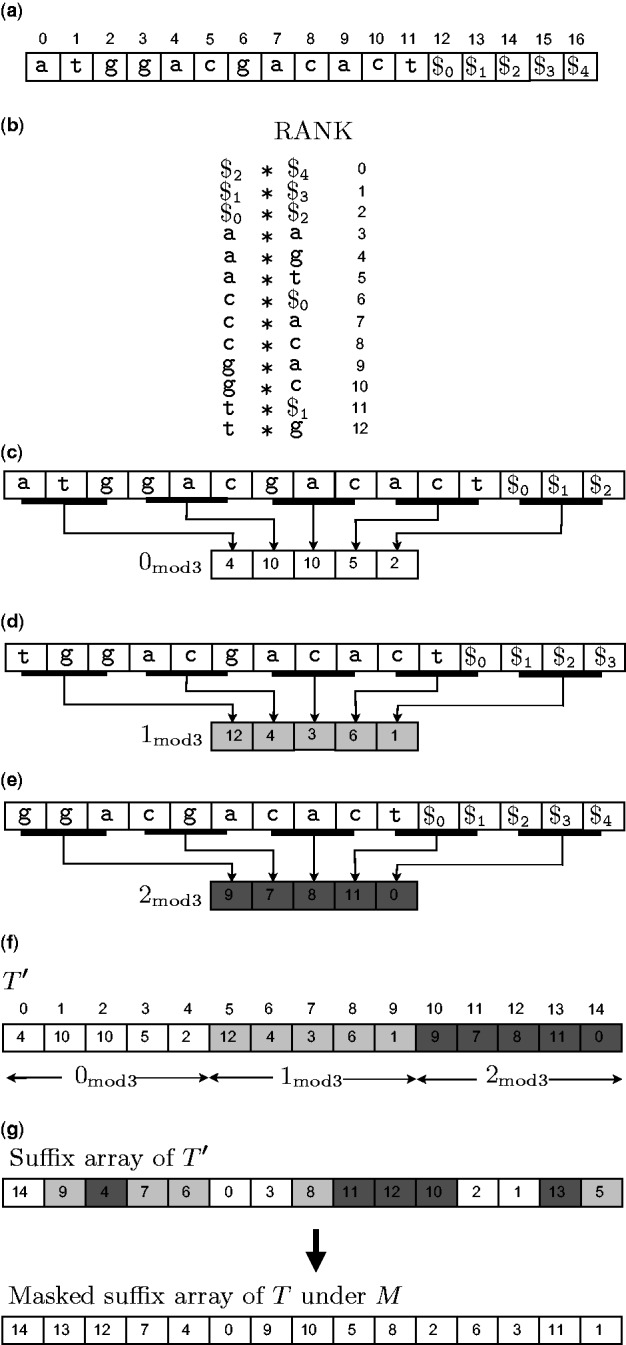
A demonstration of how DisLex constructs a spaced suffix array using an example string 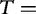 atggacgacac$ and mask 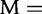 101. The characters at the 0 positions of the mask have been mapped to the character 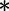. (a) The input string with extra padding. (b) Lexically sorting all the length-3 distinct substrings of *T*. The mapping *RANK* is defined using this ordering. (c), (d), (e) Constructing 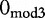, 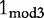 and 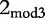, respectively. (f) Constructing 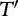 by concatenating 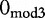, 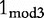 and 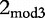. (g) The suffix array of 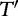 (above) is transformed to the spaced suffix array of *T* (below).

In Figure 10, we illustrate steps 1 and 2 with the running example of 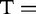 atggacgacact with 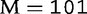.

##### Transforming T to 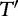


Assume *T* does not contain any sentinel character, and for the sake of simplicity, suppose the length of *T* is some multiple *k* of the mask length 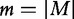. Append at the end of *T* a string 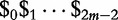 of 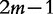 sentinel characters (if *T* is not an exact multiple of *m*, extra sentinel characters can be added to make it so). The extra characters are lexically smaller than any character of *T*, and among themselves are related as 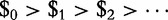 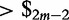. The relevance of this padding will be apparent later. From here onwards, *T* refers to this padded string of length of 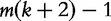.

Take all distinct length-*m* masked substrings of *T* and compute their lexical ordering, for example by radix sorting. Let us define a mapping RANK that maps each distinct masked substring to its rank in this ordering (Figure 10b).

Next construct a string 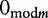 from 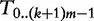 by replacing the length-*m* length substrings 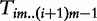, 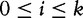 by RANK(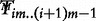) (Figure 10c). The reason behind this rather peculiar naming is that the suffixes of 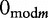 correspond to those masked suffixes of *T* whose index position *i* has the property that 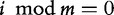. In a similar fashion of lexically naming length-*m* substrings, construct string 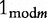 from 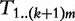, string 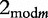 from 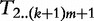, and so on up to 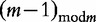 from 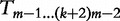 (Figure 10d, e). Finally, concatenate 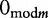, 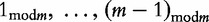 to obtain the DisLex text 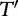 (Figure 10f).

##### Reverse transformation

Step 2 produces the ordinary suffix array of 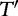. The spaced suffix array of *T* can be easily computed using the one-to-one correspondence between the suffixes of 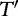 and the masked suffixes of *T*. Let us see how to describe this correspondence in more mathematical terms.

Consider a suffix 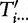 of 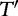. If we think of 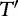 in terms of the *m* blocks 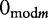 to 
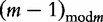
 each *k* + 1 long, then the position index *i* can be expressed as 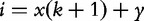 for some integer *x* 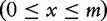 and some integer *y* 
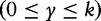
—the value *x* indicates which block 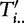 starts in and *y* tells us its position offset within this block. Then the index of the corresponding masked suffix of *T* is 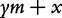. For example in (Figure 10(f,g)), 9 in the suffix array of T' corresponds to 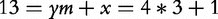 in the masked suffix array of T. In this computation, 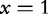 because 9 is in the mod1 block of T' 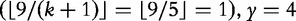 because 9 mod 5 = 4.

##### Correctness

Why does sorting suffixes of 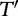 correspond to sorting masked suffixes of *T*? It again helps to think of 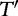 as a concatenation of the blocks of strings 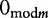 to 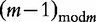. Consider a pair of masked suffixes 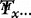 and 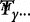 of *T*. Suppose 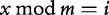 and 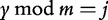 (with the possibility that *i* = *j*), then the suffix of 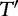 corresponding to 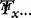 starts in the 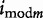 block, and that corresponding to 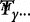 starts in the 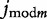 block. If we look at just the strings 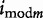 and 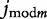 (i.e. isolated from 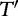), then we can see that our lexical naming technique ensures that the lexical relation between 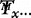 and 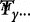 is exactly the same as that between their corresponding suffixes in 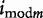 and 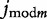. However, in 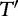, there could be other characters following 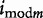 and 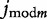. This is where the role of the padding comes in. We padded *T* with enough sentinel characters so that the last character of each block receives a delimiter-type lexical name (lexically less than any name not involving sentinel characters). Therefore, it does not matter that in 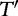, blocks 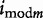 and 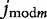 are followed by other characters.

##### Computation time

The running time of the DisLex transformation is 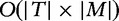 time. In practice, 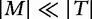 and the time needed for the DisLex transformation and reverse transformation is much less than that needed for the subsequent ordinary suffix array construction in Step 2. The results of simple experiments with human Chromosome 1 and two masks are shown in Table 2.

**Table 2:** Running time (in seconds) of the three steps of DisLex with human Chromosome 1 (∼225 million characters) as input, using the ‘codon mask’ (101) and a mask used by PatternHunter [44] (111010010100110111)

Mask	Time in seconds		
	Step 1	Step 2	Step 3
101	2	123	2
*PatternHunter*	4	213	2

The suffix array of the LexText in step 2 is constructed with SA-IS, using code by P.H. based on Ge Nong’s SA-IS implementation.

The main practical drawback to DisLex is that, depending on the mask, the alphabet size of the DisLex text may in general become quite large (although always bounded by 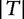), even when the original alphabet size is small. The ‘codon mask’ 101 is relatively innocuous in this respect, as it has only two care positions and therefore at most squares the original alphabet size (ignoring the small overhead due to sentinel characters). Many highly sensitive seeds are, however, much longer and contain many more care positions, which can result in a large alphabet. Recalling the issues raised in section ‘Computational Complexity’ and analyzed in depth in section ‘Memory usage of SA-IS’ in the supplementary material, we can see that an increased alphabet size increases memory use in two ways: (i) depending on the mask, the DisLex text may require 4 bytes per character, instead of one; and (ii) when SA-IS is used as the suffix array construction algorithm in Step 2, a large number of bucket pointers may be needed to induced-sort *T*.

### Subset seeds

The concept of subset seeds is a generalization of spaced seeds. With spaced seeds, any kind of mismatches are allowed at 0 positions of the mask. With subset seeds, we can specify the types of mismatches that are allowed at each position.

For instance, at some positions we might want to make no distinction between the two purines 
a,g or between the two pyrimidines 
c,t—the rationale being that transition mutations have a higher frequency than transversions. For protein sequences, we might want to allow mismatches between similar amino acids. It has been shown that a carefully chosen pattern of subset seeds is even more effective than spaced seeds in improving the sensitivity of alignment programs [49, 50].

DNA methylation measurement via bisulfite sequencing is an important application in which the need for subset seeds is especially clear. DNA methylation is an epigenetic modification in which a methyl group is chemically added to a nucleotide (typically cytosine) by cellular methyltransferases. This phenomenon is of great interest because it plays an important role in gene expression and cellular differentiation. Treating DNA with bisulfite converts the unmethylated cytosines into uracils, but leaves methylated cytosines unchanged. In a subsequent step, PCR (in which uracil acts like thymine) is used to amplify those sequences. Thus, in bisulfite sequencing methylated cytosines appear as t’s. If we are to then use a seed-and-extend method to align these sequences to a reference genome, we need to use subset seeds that allow a c-t mismatch (g-a mismatch in the reverse strand) to account for the fact that a t in the query could have possibly originally been a c.

Like spaced seeds, we can describe a subset-seed pattern using a mask. Unlike a binary string mask used to represent spaced seeds, however, a length-*m* mask for a subset seed is an *m*-tuple 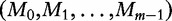, where each *M_i_* is a collection of disjoint sets, each set defining an equivalence class of characters for a particular position. For instance, 
({{a,g},{c,t}},{{a,c,g,t}},{{a}, {c},{g},{t}}) allows 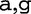-mismatch and 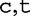-mismatch in the first position, any kind of mismatch in the second and only exact matches in the third. Thus, applying a subset seed mask to a string can be thought of as replacing equivalent characters at each position by some fixed character. As with spaced seeds, subset seeds are applied cyclically or trimmed to adjust to the length of the string being masked.

To facilitate subset-seed queries, we would like to sort the suffixes of a given text under a given mask. DisLex can easily accommodate subset seeds with a bit of modification in Step 1. When sorting the length-*m* distinct substrings to compute the mapping RANK, we must apply the subset seed mask.

### Approximate patterns based on edit/Hamming distance

Another type of string search problem is one in which given a string *T*, a short query string *P* and a positive integer *k*, we wish to find the occurrences of strings in *T* that are within Hamming or edit distance *k* from *P*. This formulation has applications for instance in mapping short reads obtained from high-throughput sequencing experiments to a reference genome. Modern-day sequencers work by first shearing the biological sample into fragments, and then sequencing the fragments. This results in billions of short DNA sequences (often called ‘reads’), which are typically 35 to a few 100 base pairs long depending on the technology. Often the first step in analyzing the enormous set of reads is to align each read to a reference genome, a task often called ‘mapping’). For shorter reads of length up to a 100 nucleotides, one popular strategy to tackle the mapping problem has been to index the reference genome, and for each read search the index to find the locations that are within a certain edit distance from the read. The edit distance threshold accounts for either genuine differences in the sample and the reference due to polymorphisms or differences due to sequencer errors. Tools like BWA [51], Bowtie [52], SOAP [53], GEM [54] and Masai [55] use this model of sequence similarity and employ index structures closely related to suffix arrays.

Theoretically suffix arrays are poorly suited for such queries. One approach is to generate the set of all strings that are within edit distance *k* of a pattern *P* and then search each of the strings in the suffix array, but this does not scale well because the set grows exponentially in the length of *P* and in *k*.

Another strategy is ‘backtracking’ in which the index is parsimoniously traversed to extract all locations of approximate matches [56]. The method also does not scale well for large *k*, but can be effective for read mapping [55].

Yet another strategy is to first filter out the locations that are highly likely to contain matches by splitting *P* into shorter substrings and finding exact matches of the substrings. To get a sense of why this works, consider searching for occurrences that are within edit distance 1 of *P*. If *P* is partitioned into nonoverlapping substrings *P*_1_ and *P*_2_, it is necessary that either *P*_1_ or *P*_2_ have an ‘exact’ match at any location that is within edit distance 1. Once the candidate locations are identified, we can then investigate them to verify if they actually contain the required matches. Again this technique performs poorly as *k* increases because the size of the partitions start getting smaller and there are too many candidate locations that need to be verified. A modified suffix array that facilitates faster identification of candidate locations of approximate matches is described in [57].

We refer the readers to [58, 59] for more detailed discussion on approximate string matching based on edit distances.

## CLOSING REMARKS

We have presented a detailed exposition of the SA-IS algorithm for suffix array construction, in the context of other linear-time algorithms and described techniques to adapt suffix arrays to inexact matching needed for bioinformatic applications. Although our exposition is by no means light reading, we believe it is considerably more accessible than the original literature.

To provide a reasonably complete and self-contained document, we intentionally limited the scope of our discussion. Thus, we chose not to treat many important topics, in particular distributed computing and storage of suffix arrays. However, just as the SA-IS algorithm builds on previous techniques, future algorithms will most likely build on techniques, such as lexical naming, reviewed in this article.

The field of genomics is rapidly developing as sequencing technology continues to advance and sequencing is applied to more and more areas outside of traditional genome sequencing, from gene expression to epigenetics and even quantitative measurement of protein translation. Patient sequencing and analysis may soon become as routine as X-rays for medical diagnosis. At the same time, computational technology is also leaping forward as our field moves towards further adoption of cloud technology and data storage moves from peta-bytes to exa-bytes and even zetta-bytes.

For an article written in such exciting times, this may seem like a rather stodgy exposition, fastidiously counting every computational operation and byte of memory used. However, we would argue that the coming deluge of sequence data will not be conquered by hardware investment alone, but rather existing and novel efficient algorithms will likely continue to be the (unsung) heroes—instrumental in realizing the potential of the sequencing revolution.

## Supplementary Data

Supplementary data are available online at http://bib.oxfordjournals.org/.

**Key Points**

- Suffix array and related data structures are indispensable to modern bioinformatics.
- Suffix arrays are used in compute-intensive applications in which their construction and memory use can be a bottleneck.
- Recently efficient and elegant (albeit somewhat complex) algorithms have been developed for efficient suffix array construction.
- Suffix arrays can be adapted to special needs in bioinformatics applications such as spaced and subset seeds in sequence homology search.

## FUNDING

This work was partially supported by a Japanese Ministry of Education, Sport, Science and Technology (MEXT) Grant-in-Aid for Scientific Research on Innovative Areas (221S002).

## Supplementary Material

Supplementary Data
