# Supplementary material for: A bioinformatician’s guide to the forefront of suffix array construction algorithms
Source: Brief Bioinform. 2014 Jan 10;15(2):138–54. doi: 10.1093/bib/bbt081 (PMC3956071; doi:10.1093/bib/bbt081)
Supplement: Supplementary Data [file supp_15_2_138__index.html]

A bioinformatician’s guide to the forefront of suffix array construction algorithms — A bioinformatician’s guide to the forefront of suffix array construction algorithms — Supplementary Data 

# A bioinformatician’s guide to the forefront of suffix array construction algorithms

## Supplementary Data

files

**Files in this Data Supplement:**

- Supplementary Data - pdf file
