# Supplementary material for: A bioinformatician’s guide to the forefront of suffix array construction algorithms
Source: Brief Bioinform. 2014 Jan 10;15(2):138–54. doi: 10.1093/bib/bbt081 (PMC3956071; doi:10.1093/bib/bbt081)
Supplement: Supplementary Data [file supp_bbt081_BiB2013_sup.pdf]

A Bioinformatician's Guide to the Forefront of Suffix Array Construction Algorithms: Supplementary Material

Anish Man Singh Shrestha[1,2], Martin C. Frith[2] & Paul Horton[2]

[1]Department of Computational Biology, Graduate School of Frontier Sciences, The University of Tokyo
[2]Computational Biology Research Center, AIST, Tokyo, Japan

S1 Supplementary material for Divide Phase of Section 3.2

S1.1 The Proper prefix problem

To describe the proper prefix problem formally it helps us to first define a *tiling* of a text T, as a set of substrings (tiles) of T such that 1) all positions in T are covered by at least one tile, 2) each position is the start of at most one tile. For example, the set of all length-two substrings is a tiling, as is the set of all length-two substrings starting at an even position (for an even length text). Note that tiles may overlap, for example in SA-IS adjacent tiles ($\vee\wedge\vee$ substrings) overlap by one position.

Now we can define *lexical naming*, a technique at the heart of all of the linear time suffix array construction algorithms. Lexical naming transforms a text T to another text T', by defining a tiling of T and treating those tiles as characters of the new text T'. The particular character used to represent a tile is chosen to reflect the rank of that tile in a lexically sorted list of the tiles. In this way every suffix $T'_{i'\cdots}$ of T' corresponds to a suffix $T_{i\cdots}$ of T. One key property that must be maintained by lexical naming is that for any two suffixes $T'_{i'\cdots}$ and $T'_{j'\cdots}$ of T',

$$T'_{i'\cdots} < T'_{j'\cdots} \iff T_{i\cdots} < T_{j\cdots}.$$

If lexical naming is done on fixed-length substrings, it is rather straightforward that the above relationship is maintained by simply using the rank of each substring.

However with variable-length substrings, as is with the case of $\surd\!\diagdown\!\surd$ substrings, this is not immediate. For example, consider $T =$ `ctsmctsy$`, and assume that `ctsm` and `cts` are two among other substrings for which we need to compute lexical names. Note that one is a proper prefix of the other. Since `ctsm` $>$ `cts`, we might want the lexical naming to assign a larger character to the former. However in T, the corresponding suffixes $T_{0\cdots}$ and $T_{4\cdots}$ have the opposite relationship, and clearly such a naming strategy will not work.

In the SA-IS algorithm, this problem goes away because of the way the $\surd\!\diagdown\!\surd$ substrings and their ordering are defined. With some thought one can see that for any $\surd\!\diagdown\!\surd$ substring pair (p, r) in which p is a proper prefix of r, the final character of p is always $\nearrow$ and the corresponding character of r is always $\searrow$. With the extra rule of checking the types, p will always be given a lower lexical name than r, as it should be (since the suffix starting at the first position of p is lexically larger than the suffix starting at the first position of r).

S1.2 Correctness of instance reduction

Here we will show that sorting the suffixes of T' corresponds to sorting the $\surd$ suffixes of T. Let us first prove the following useful lemma.

Lemma S1. *Between a pair of $\searrow$-type and $\nearrow$-type suffixes, both starting with the same character, the $\searrow$ suffix is lexically smaller than the $\nearrow$ one.*

Proof. Let I and J be two suffixes starting with the same character c, but of types $\searrow$ and $\nearrow$, respectively. Consider the first position where they have different characters. In both I and J, all the characters leading up to this position must also be c (otherwise it cannot be that I and J are of different types). At the position where they differ, given their types, I must have a character smaller than c, whereas J must have a character larger than c. Thus $I < J$. □

Theorem S1. *Sorting the suffixes of T' corresponds to sorting the $\surd$ suffixes of T.*

Proof. We defined T' such that each of its character corresponds to a $\surd\!\diagdown\!\surd$ substring; and as a consequence, each $\surd$ suffix $T_{i\cdots}$ of T corresponds to a suffix of T', which we will refer to as $T'_{i'\cdots}$. We need to show that the lexical relation between any two suffixes $T'_{i'\cdots}$ and $T'_{j'\cdots}$ of T' is the same as that between their corresponding suffixes $T_{i\cdots}$ and $T_{j\cdots}$ of T, i.e.

$$T'_{i'\cdots} < T'_{j'\cdots} \iff T_{i\cdots} < T_{j\cdots}.$$

First let us show that $T'_{i'\cdots} < T'_{j'\cdots} \implies T_{i\cdots} < T_{j\cdots}$. Suppose $T'_{i'\cdots} < T'_{j'\cdots}$. Since a common prefix between $T'_{i'\cdots}$ and $T'_{j'\cdots}$ implies a common prefix between $T_{i\cdots}$ and $T_{j\cdots}$, let us consider the position at which $T'_{i'\cdots}$ and $T'_{j'\cdots}$ disagree first. Let I be the

$\surd\!\wedge\!\surd$ substring of T corresponding to this position in $T'_{i' \cdots}$, and let J be the $\surd\!\wedge\!\surd$ substring corresponding to that in $T'_{j' \cdots}$. Since $T'_{i' \cdots} < T'_{j' \cdots}$, it must be that according to our definition of the ordering between two $\surd\!\wedge\!\surd$ substrings, $I < J$. Recall that we had defined this ordering as follows: if the characters under comparison are different, then we apply the usual lexical rule; and if they are the same, then we look at their types, with $\nearrow$-type character defined to be larger than $\searrow$-type. Therefore we distinguish two cases.

Case 1: The first position in which I and J differ have different characters. Then at that position, the character in I is smaller than the one in J. This implies that $T_{i \cdots} < T_{j \cdots}$.

Case 2: The first position in which I and J differ have the same character in both I and J, but the character in I is $\searrow$ type and the one in J is $\nearrow$ type. Thus we have from Lemma S1 that $T_{i \cdots} < T_{j \cdots}$.

We can show that $T'_{i' \cdots} > T'_{j' \cdots} \implies T'_{i' \cdots} > T'_{j' \cdots}$, using the same flow of logic as above. □

S1.3 Sorting the substrings

In this subsection, we will prove the following theorem.

Theorem S2. *The $\surd\!\wedge\!\surd$ substrings can be sorted in time linear to the length of T.*

S1.3.1 The Method

Following Nong et al., let us first define a few new terms. For every suffix $T_{i \cdots}$ of T, its $\surd$-prefix is the string $T_{i..j}$, where $j > i$ and $T_{j \cdots}$ is the first $\surd$ suffix to start at a position to the right of T_i. Additionally, the sentinel is defined to be a $\surd$-prefix. A $\surd$-prefix is also classified as $\nearrow$ or $\searrow$ depending on the type $T_{i \cdots}$ is. Note that by this definition, a $\surd\!\wedge\!\surd$ substring is also a $\nearrow$-type $\surd$-prefix. Similar to the ordering of $\surd\!\wedge\!\surd$ substrings, we will define the ordering of $\surd$-prefixes based on both character and type (i.e. the order is first determined by the character, and if they are equal by the types, $\nearrow$ being smaller than $\searrow$. We will (ab)use the $\prec$, $\succ$, $\approx$ symbols to denote the relationship between two $\surd$-prefixes based on this ordering, so that they are distinguished from the regular $<$, $>$, $=$ that we have reserved for the normal lexical ordering of full-length suffixes.

What makes SA-IS so interesting is that sorting $\surd\!\wedge\!\surd$ substrings is almost exactly the same as the 3-step combine phase (main article Section 3.2.3). It might be beneficial to read that section before proceeding. To recall, the combine phase uses the ordering of $\surd$ suffixes to order the $\searrow$ suffixes; and then uses the order of the $\searrow$ suffixes to sort the $\nearrow$ suffixes. In contrast, to sort the $\surd\!\wedge\!\surd$ substrings, first the $\searrow$-type

$\surd$-prefixes are sorted based on their end $\surd$ characters; and then the $\nearrow$-type $\surd$-prefixes are sorted based on the ordering of the $\searrow$-type $\surd$-prefixes. Since $\surd\backslash\surd$ substrings are also $\nearrow$-type $\surd$-prefixes, they end up being sorted too.

We will now describe the method in more detail, and then follow it with a proof of correctness.

Let A be an array of the size of T, which we will use to sort the $\surd$-prefixes. As in the combine phase, A can be thought of as being partitioned into buckets depending on the first character of the $\surd$-prefixes. Each bucket can then be divided into two sub-buckets: the left one for $\searrow$ type and the right one for $\nearrow$ type. This is because Lemma S1-like relationship holds for $\surd$-prefixes also. There is one distinction however: two $\surd$-prefixes can be equal, and we allow them to occupy neighboring positions in A in any order.

Step 0: This step initializes A and places the $\surd$-type suffixes in their appropriate buckets. Set all elements of A to the special value of -1. Compute the $\nearrow$-type bucket boundaries of A and save those in an array of tail pointers. Place each $\surd$-type suffix (precisely speaking, its index into T) at the end of the $\nearrow$-type bucket then decrement that tail pointer to point one position to the left.

Step 1: This step orders the $\searrow$-type $\surd$-prefixes based on the order of the $\surd$ suffixes (or rather the $\surd$ characters) from the earlier step. Scan A from left to right, skipping any elements with value -1. For each suffix index A_i encountered, if T_{A_i-1} is $\searrow$-type, place $A_i - 1$ at the current head of its respective bucket, and then increment that head pointer.

Step 2: This step uses the order of the $\searrow$-type $\surd$-prefixes obtained from Step 1 to sort the $\nearrow$-type $\surd$-prefixes. Scan A from right to left. For each suffix index A_i encountered, if T_{A_i-1} is $\nearrow$-type, place A_{i-1} into the current tail of its respective bucket and decrement that tail pointer.

S1.3.2 Proof of Correctness

Before we begin a formal proof of correctness, we make two observations regarding step 0. The first is that the order in which the $\surd$-prefixes is places does not matter, as long as they are placed in their appropriate buckets. The second is that by doing this, the length-one $\surd$-prefixes are correctly prefix sorted. Thus the situation is highly analogous to the combine phase of the main algorithm in which the $\surd$-suffixes? are assumed to be correctly sorted via recursion.

The following lemma is not difficult to see.

Lemma S2. *For a $\searrow$-type $\surd$-prefix $T_{i..j}$, $T_{i..j} \succ T_{i+1..j}$.*

Lemma S3. *At the end of Step 1, the $\searrow$-type $\surd$-prefixes are in their correct positions.*

Proof. We will show this by proving the following proposition: when the sweep reaches the i-th position of the array A, every position between 0 to i that is inside a $\searrow$-type bucket already contains the correct $\searrow$-type $\surd$-prefix in it. The proof is by induction on i.

For $i = 0$, this statement is vacuously true because the 0-th position is not in a $\searrow$ bucket, rather it contains $T_{n-1..n-1}$ from Step 0.

Assuming that the proposition is true for some $i > 0$, we will show that it is also true for $i + 1$. Suppose the contrary. Since the $\searrow$-type $\surd$-prefixes up to i have been correctly placed and we don't move them once placed, it must be that the $(i + 1)$-th position has an incorrect $\searrow$-type $\surd$-prefix. Let $T_{j..k}$ be this incorrectly placed $\surd$-prefix, and let $T_{l..m}$ be the correct one that should have been placed there. It must be that $T_{j..k} \succ T_{l..m}$. This is because if $T_{j..k} \approx T_{l..m}$, then it has been correctly placed; and if $T_{j..k} \prec T_{l..m}$, it would have been already placed in a position somewhere left of $i + 1$ by the induction hypothesis. Also both $T_{j..k}$ and $T_{l..m}$ start with the same character because they were placed in the same bucket.

Let us have a look at $T_{l+1..m}$ and $T_{j+1..k}$. Since T_l is $\searrow$ type, we have from Lemma S2 that $T_{l+1..m} \prec T_{l..m}$. Then it must be that $T_{l+1..m}$ is already placed in A in some position left of $i + 1$ because of one of the following:

Case 1: if $T_{l+1..m}$ is $\searrow$-type $\surd$-prefix, by induction hypothesis.

Case 2: if $T_{l+1..m}$ consists of a single $\surd$-type character, it must be lexically smaller than T_l; and therefore in Step 0, it was placed in its the bucket, which must be left of $i + 1$.

As for $T_{j+1..k}$, it can either be left of $i+1$ or not. If the latter is true, then it cannot be the case that $T_{j..k}$ got inserted before $T_{l..m}$ because the left-to-right sweep would have reached $T_{l+1..m}$ first. Suppose the former is true. Since we established that $T_{j..k}$ and $T_{l..m}$ start with the same character and that $T_{j..k} \succ T_{l..m}$, we have $T_{j+1..k} \succ T_{l+1..m}$. Using this, we can show that $T_{l+1..m}$ is left of $T_{j+1..k}$ because of one of the following:

Case 1: if both $T_{j+1..k}$ and $T_{l+1..m}$ are $\searrow$-type $\surd$-prefixes, then by induction hypothesis.

Case 2: if both of them consist of single $\surd$-type character, then they are in different buckets in Step 0. (Note that they cannot be in the same bucket, else $T_{j..k} \approx T_{l..m}$.)

Case 3: if one of them is $\searrow$ type, and the other $\nearrow$ type, then the buckets they belong to already ensures this relative positioning.

Since $T_{l+1..m}$ is left of $T_{j+1..k}$, the sweep would have reached $T_{l+1..m}$, first and therefore this contradicts our assumption that $T_{j..k}$ was inserted before $T_{l..m}$. This completes the proof that Step 1 correctly orders the $\searrow$-type $\surd$-prefixes. $\square$

Lemma S4. *At the end of Step 2, the $\nearrow$-type $\surd$-prefixes are in their correct positions.*

Proof. Similar to the proof above, we will show this by proving the following proposition: when the sweep reaches the i-th position of the array A, every position between i to $n-1$ contains the correct $\surd$-prefix in it.

The proof is by induction on i.

The base case is that of $i = n - 1$. The $\surd$-prefix in that position in A has to be an $\searrow$-type one because we cannot have an $\nearrow$-type $\surd$-prefix starting with the largest alphabet. From the previous lemma, it must be in its correct position.

Next we need to show that if the proposition is true for some $i < n - 1$, it is also true for $i-1$. Since the proof is similar to that of the lemma above, we will skip it. □

The time complexity of this method is $O(n)$, as each step is a sweep across the length-n array A, with each operation along the sweep requiring $O(1)$ time.

Thus we have proved Theorem S2.

S1.4 Computing the reduced instance T' from the ordering of the substrings

One minor thing that remains to be discussed is how to obtain the reduced instance T' from this ordering of the $\surd\!\wedge\!\surd$ substrings. We will show how to construct T' in-place in A, when we discuss the memory usage of SA-IS in Section S3.

S2 Supplementary material for Combine Phase of Section 3.2

S2.1 Correctness of Step 1 of combine phase

Theorem S3. *At the end of Step 1, all $\searrow$-type suffixes are in their correct positions.*

Proof. We will show this by proving the following proposition: when the scan reaches the i-th position of the array A, every position between 0 to i that is inside a $\searrow$-type bucket already contains the correct suffix in it.

The proof is by induction on i.

For $i = 0$, this statement is vacuously true because the 0-th position is not in a $\searrow$ bucket, rather it contains $T_{n-1\cdots}$ from Step 0.

Assuming that the proposition is true for some $i \geq 0$, we will show that it is also true for $i + 1$. Suppose the contrary. Since the $\searrow$-type suffixes up to i have been correctly placed and we don't move them once placed, it must be that the $(i + 1)$-th position has an incorrect suffix. Let this suffix be $T_{j\cdots}$ for some j. Note that $T_{j\cdots}$ must

be $\searrow$ as well, because we do not insert any $\nearrow$-type suffixes in Step 1. Let $T_k \ldots$ (for some $k \neq j$) be the correct $\searrow$ suffix that should have been placed at $i+1$. It must be that $T_j \ldots > T_k \ldots$ because otherwise $T_j \ldots$ would already be in its correct position by the induction hypothesis. Also since $T_j \ldots$ was placed in the $\searrow$-type bucket in which $T_k \ldots$ should have been, they must have the same initial character.

Let us have a look at the suffixes $T_{k+1} \ldots$ and $T_{j+1} \ldots$. We have $T_{k+1} \ldots < T_k \ldots$ since the latter is $\searrow$ type. Then it must be that $T_{k+1} \ldots$ is already placed in A in some position left of $i+1$ because of one of the following:

Case 1: if $T_{k+1} \ldots$ is $\searrow$ type, by the induction hypothesis.

Case 2: if $T_{k+1} \ldots$ is $\surd$ type, the starting character of $T_{k+1} \ldots$ is lexically smaller than the starting character of $T_k \ldots$; and therefore the bucket for this $\surd$ type is to the left of $i+1$.

As for $T_{j+1} \ldots$, it can either be to the left of $i+1$ or not. If the latter is true, then it cannot be the case that T_j was inserted before T_k because the scan proceeds from left to right and would have encountered $T_{k+1} \ldots$ first. Suppose the former is true. Since we already established that $T_j \ldots$ and $T_k \ldots$ have the same initial character and that $T_j \ldots > T_k \ldots$, we have $T_{j+1} \ldots > T_{k+1} \ldots$. Using this, we can show that T_{k+1} is left of T_{j+1} because:

Case 1: if both $T_{j+1} \ldots$ and $T_{k+1} \ldots$ are $\searrow$ type, by the induction hypothesis.

Case 2: if both $T_{j+1} \ldots$ and $T_{k+1} \ldots$ are $\surd$ type, they have been inserted in A in their sorted order in Step 0.

Case 3: if one of them is $\searrow$ type and the other $\surd$ type, the buckets they belong to ($\searrow$ and $\nearrow$ respectively) ensure this relative positioning.

Thus the scan would have encountered $T_{k+1} \ldots$ before $T_{j+1} \ldots$, and therefore it cannot be the case that T_j was inserted before T_k. Therefore our initial assumption that $i+1$ does not have the correct suffix is wrong. This completes the proof by induction. □

S2.2 Correctness of Step 2 of combine phase

Theorem S4. *At the end of Step 2 of Combine Phase, all the suffixes are in their correct position.*

Proof. The $\searrow$ suffixes were placed in their correct position by Step 1, and we do not move them in Step 2. Therefore we only need to show that this step places all the $\nearrow$ suffixes in their correct positions. We will show this by proving the following proposition: when the right-to-left scan reaches the i-th position of the array A, every position between i and $n-$ that is inside a $\nearrow$-type bucket already contains the correct suffix in it. The proof is by induction on i. The base case is that of $i = n-1$. The suffixes starting from the largest alphabet are always $\searrow$ type. Because Step 1 places

all $\searrow$-type suffixes in the correct position, the suffix at position $n-1$ is $\searrow$. Therefore the base case is vacuously true.

The induction phase is very similar (in fact simpler) than the proof of Theorem S3, so we will skip it. □

S3 Memory usage of SA-IS

We mentioned in Section 4.2 that the SA-IS algorithm requires about $2n$ bytes of working memory for a text T of length n. This is under the assumption that each character can be represented by 1 byte and that each position index of the suffix array can be represented by a 4-byte integer (this limits the text size to $< 2^{32}$). In this section, we will provide a thorough analysis of the space requirement of SA-IS. We begin by considering a rather straightforward implementation in order to understand where memory costs are incurred, and then show how to improve this implementation using simple but (perhaps) non-obvious techniques.

S3.1 Simple Implementation

Suppose the computation has reached some level i of recursion (level 0 being the topmost). The memory requirement at level i depends on whether we have arrived at this level from the shallower level $i-1$ on the way down the recursion path (in which case the divide phase is executed), or from the deeper level $i+1$ on the way up (in which case the combine phase is executed). Let us treat these two cases separately.

Memory usage of the divide phase at level i

In this section, we denote the text at level i of recursion as T_i and its length by n_i, with $n_0 = n$. As detailed in Section 3.2.1 of the main article and Section S1.3 above, SA-IS does the following computations in the divide phase: (1) classify the suffixes as $\nearrow$ or $\searrow$ type, (2) induced-sort the $\surd\!\!\diagdown$ substrings, and (3) generate the reduced instance T_i' which serves as the input text T_{i+1} for the next level of recursion. The major memory allocations required for these computations are as follows:

- an array TYPE_i of size n_i bits indicating the type of each suffix,
- an array A_i of $4n_i$ bytes to induced-sort the $\surd\!\!\diagdown$ substrings,
- an array B_i of size equal to the number of distinct characters in T_i, to hold the bucket pointers required for induced-sorting of the $\surd\!\!\diagdown$ substrings (recall that the bucket heads and tails are never needed in the same step so only one pointer per character is required).

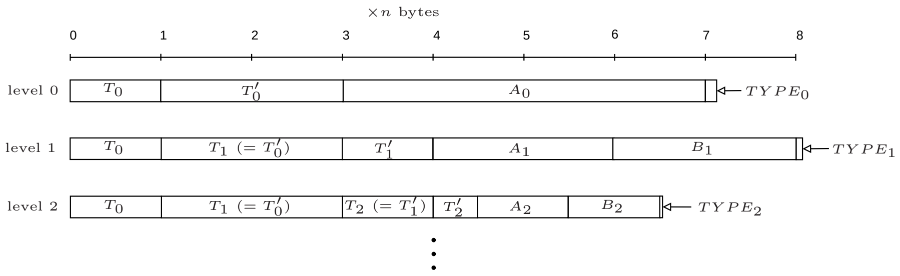

Figure S1: Each horizontal bar represents the worst-case (T_{i+1} is half the size of T_i and when B_i is in the order of the size of T_i) peak memory usage for the divide phase at a particular recursion level. B_0 is not shown as it is of some constant size, assuming T_0 is a text over a constant alphabet.

- an array to hold the reduced text T_i' that is constructed by lexical-naming the √∨ substrings.

Before the computation proceeds to the next level of recursion, we can discard TYPE$_i$, B_i, and A_i from memory. The first two will be required again when we revisit level i on the way back from level $i+1$, but we can generate them from T_i when that need arises.

Based on the observations above, we show in Figure S1 the worst-case memory requirement at different levels of recursion. The maximum is at level 1, with a footprint of $8n$ bytes plus $n/2$ bits.

Memory usage of combine phase at level i

We now turn our attention to the case where the computation returns to level i from level $i+1$. Level $i+1$ returns $SA^{T_{i+1}}$, the suffix array of T_{i+1}. The suffixes in $SA^{T_{i+1}}$ correspond to the √ suffixes of T_i, and as detailed in Section 3.2.3 of the main article, the suffix array of T_i can be computed using this information by induced-sorting. The following memory allocation is required:

- an array TYPE$_i$ of size n_i bits indicating what type each suffix is,
- an array A_i of size $4 \times n_i$ bytes to induced-sort suffixes of T_i and to ultimately hold its suffix array,
- an array B_i of size equal to the number of distinct characters in T_i to hold the bucket pointers required during induced-sorting of the suffixes of T_i.

Note that T_{i+1} loses its utility once its suffix array is computed, and therefore it need not be passed on to level i. Also the bucket and the type arrays can be discarded before proceeding to a shallower level.

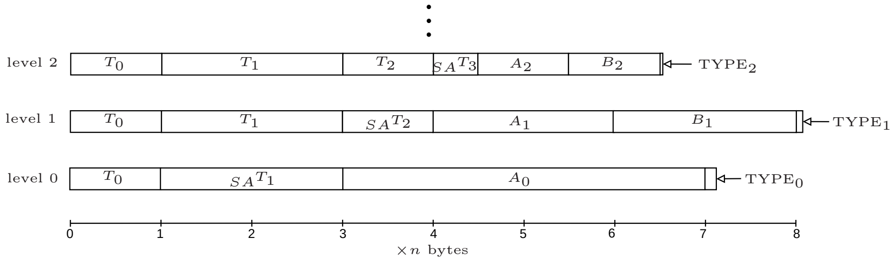

Figure S2: Worst-case memory requirement for combine phase at different levels of recursion. B_0 is not shown as it is of some constant size, assuming T_0 is a text over a constant alphabet.

Figure S2 shows the worst-case memory usage for the combine phase at different levels of recursion. Again, the maximum of $8n$ bytes plus $n/2$ bits is reached at level 1.

Thus the working memory of this simple implementation is roughly $3n$ bytes plus $n/2$ bits. We next show how to bring this figure down to $2n$ bytes by being a bit more frugal during both the combine and divide phases. While a reduction of n bytes might not sound big, mammalian genome sizes just happen to be on the same order of magnitude as modern memory sizes, so saving n bytes can in some cases have significant practical ramifications.

S3.2 Improving memory usage

The key idea in reducing the working memory to $2n$ is to reuse slices of the $4n$-byte array A_0 (the space allocated at level 0 to induced-sort the $\sqrt{\vee}$ substrings) at all subsequent levels. Since A_0 will also ultimately hold the suffix array of the input text, this $4n$ bytes does not count towards working memory. The only major working memory allocation will be for the bucket pointers. The following subsections carve out the details of this implementation idea. Again, we will split the discussion into the divide and combine phases.

Improving divide phase

In the simple implementation described in the previous section, a fresh memory allocation for arrays A_i and T_i' was made at each level i. Instead, A_i can use half the space previously used by A_{i-1} (for $i = 0$, $4n$ bytes are freshly allocated), and T_i' can be constructed in-place in A_i. This memory allocation scheme is better explained by Figure S3, from which it is clear that the divide-phase worst-case working memory is now solely dominated by the (at most) $n/2 \times 4$ bytes of the bucket pointers.

It remains to see how T_i' can be constructed in-place in A_i. We describe the process

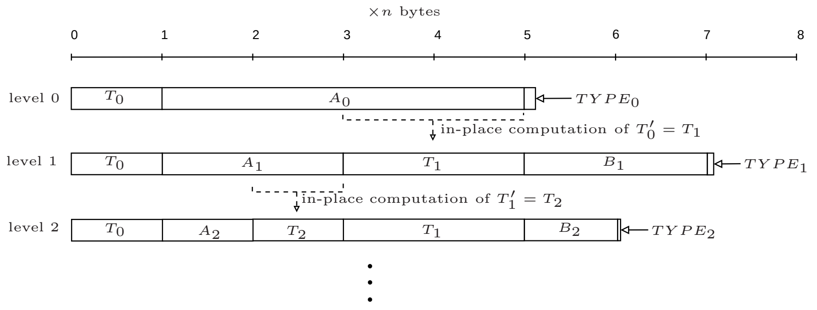

Figure S3: Memory requirement with improved implementation of divide phase. We begin by allocating $4n$ bytes for A_0. After A_0 is used for induced sorting, T_0' is constructed in-place in A_0 such that at the end of its construction, it occupies the right half of A_0. The left half of it is used by A_1 at Level 1. This pattern is repeated in subsequent recursion levels.

with a running example in Figure S4. We can think of A_i in terms of its two halves (let's call the left half A_i^l and the right A_i^r). After the induced-sorting computation is over, A_i contains the indices of $\surd$-prefixes in their sorted order. Since we are only interested in the indices of the $\surd$-prefixes that constitute $\surd\!\diagdown\!\surd$ substrings, we can pack them into A_i^l. This is possible since there at most $\lfloor |A_i|/2 \rfloor$ suffixes of type $\surd$ in T_i. We now have A_i^r free for constructing T_i'.

A_i^l helps us compute T_i' in two ways; both to compute the lexical names of the $\surd\!\diagdown\!\surd$ substrings and where they should appear in T_i'. To compute the lexical names first prepare a lexical name counter with an inital value of zero. We then scan A_i^l from left to right, using T_i to check if each $\surd\!\diagdown\!\surd$ substring is distinct (type considered) from the $\surd\!\diagdown\!\surd$ substring encountered immediately beforehand in the scan, and if so we increment the lexical name counter. We then place the current lexical name at position $\lfloor \frac{j}{2} \rfloor$ of A_i^r, where j is the value of A_i^l of the current position of the scan. Because neighboring suffixes in T_i cannot both be of type $\surd$, dividing by two never leads to a collision. Finally, we can remove the empty items between the lexical names to obtain T_i'.

It goes without saying that if all the lexical names are distinct, we can directly compute the suffix array of T_i' without further recursion and place it in A_0^l.

Improving combine phase

Now let us look at the combine phase at level i. Consider the slice of A_0 spanning its first $1/2^i$ positions, and let's call it A_i. From the scheme of partitioning A_0 in the divide phases of the deeper levels, the right half of A_i (A_i^r) contains T_{i+1}. Assuming that the first $|T_{i+1}|$ positions of the left half of A_i (A_i^l) hold the suffix array of T_{i+1}, we can execute the combine phase such that the suffix array of T_i is computed in-place in A_i. The profile of memory use at different levels of recursion is shown in Figure S5,

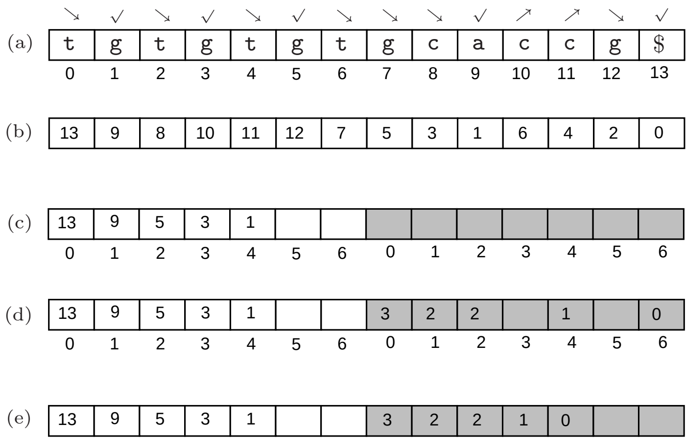

Figure S4: An example of constructing T_i' in-place in A_i. Here $i = 0$. (a) Text T_0 (b) A_0 after induced sorting of $\surd$-prefixes. (c) A_0^l (the non-shaded half) now packs the indices of T_0 corresponding to $\surd$ suffixes. (d) Lexical names are inserted into A_0^r such that they are in the order in which they should appear in T_0'. (e) The lexical names are packed to the left of A_0^r to obtain T_0'.

from which we can see that the peak working memory is $2n$ bytes plus $n/2$ bits.

We now describe how to implement the combine phase in-place, along with a running example in Figure S6. $SA^{T_{i+1}}$ which is inside A_i^l contains the suffixes of T_{i+1}, and therefore we first replace them by the corresponding suffixes of T_i. Recall that the j-th suffix of T_{i+1} corresponds to the j-th $\surd$ suffix of T_i. We can easily work out the correspondence between the indices, by making use of A_i^r. We first clear T_{i+1} off A_i^r as it is no longer needed, and use it instead to list the $\surd$ suffixes of T_i in their order of appearance in T_i. Then for every suffix j in A_i^l , we replace it with the j-th suffix in A_i^r.

Now that A_i^l contains the $\surd$ suffixes of T_i in their sorted order, we prepare A_i (specifically the first $|T_i|$ positions) for the induced-sorting phase. This means that the bucket boundaries need to be computed, and that the $\surd$ suffixes have to be placed inside their respective buckets in A_i. The latter can be done by simply scanning the suffixes in A_i^l from right to left, and for every suffix encountered, placing it inside its respective bucket. But A_i^l is part of A_i, and we have to make sure that we don't end up overwriting a position of A_i^l before having scanned it. In fact, this is guaranteed. Assume otherwise, and suppose that when the scan has only reached $A_i^l[p]$, we attempt to insert a suffix S at $A_i[q]$, where $q < p$. This would mean that there are q suffixes lexically smaller than S; but also at the same time, there are p suffixes of type $\surd$ lexically smaller than S, a clear contradiction. Once the $\surd$ suffixes are in place, we can proceed with induced-sorting (Steps 1 and 2 of the combine phase).

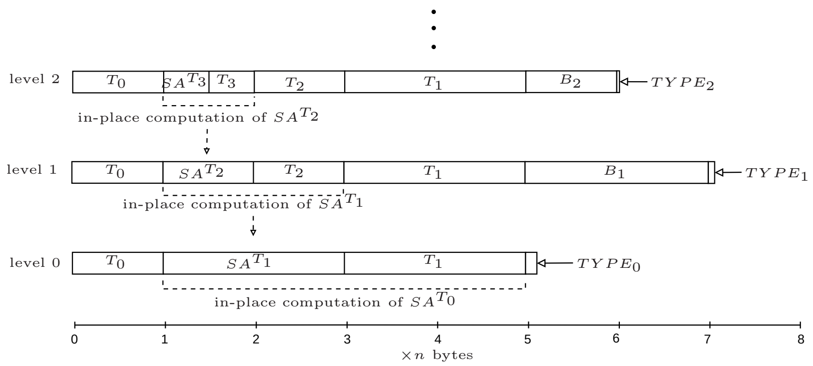

Figure S5: Worst-case peak memory requirement for improved combine phases at different levels. Compared with Figure S2, we can see that n bytes are saved.

We conclude this section by noting that since both the improved combine and divide phases require in the worst case $2n$ bytes plus $n/2$ bits, this figure summarizes the overall memory usage of SA-IS. Note that $n/2$ is an upper bound on the maximum number of bucket pointers needed. The precise maximum number of bucket pointers needed is a function of the input string and in general may be considerably less than $n/2$.

S4 Suffix array construction programs used for benchmarking tests

In the benchmarking tests in Section 4.3 of the main article, we tested several suffix array construction programs available on the internet. The links to the implementations are shown in the Table S1 below.

Implementation:	Available at:
SA-IS by Yuta Mori	`https://sites.google.com/site/yuta256/sais`
SA-IS by Nong et al.	`http://code.google.com/p/ge-nong/`
SACA-K by Nong et al.	`http://code.google.com/p/ge-nong/`
Deep Shallow by Manzini et al.	`http://people.unipmn.it/manzini/lightweight/`
lastdb (radix sort) by Frith et al.	`last.cbrc.jp`

Table S1: Suffix array construction software used for our benchmarking tests.

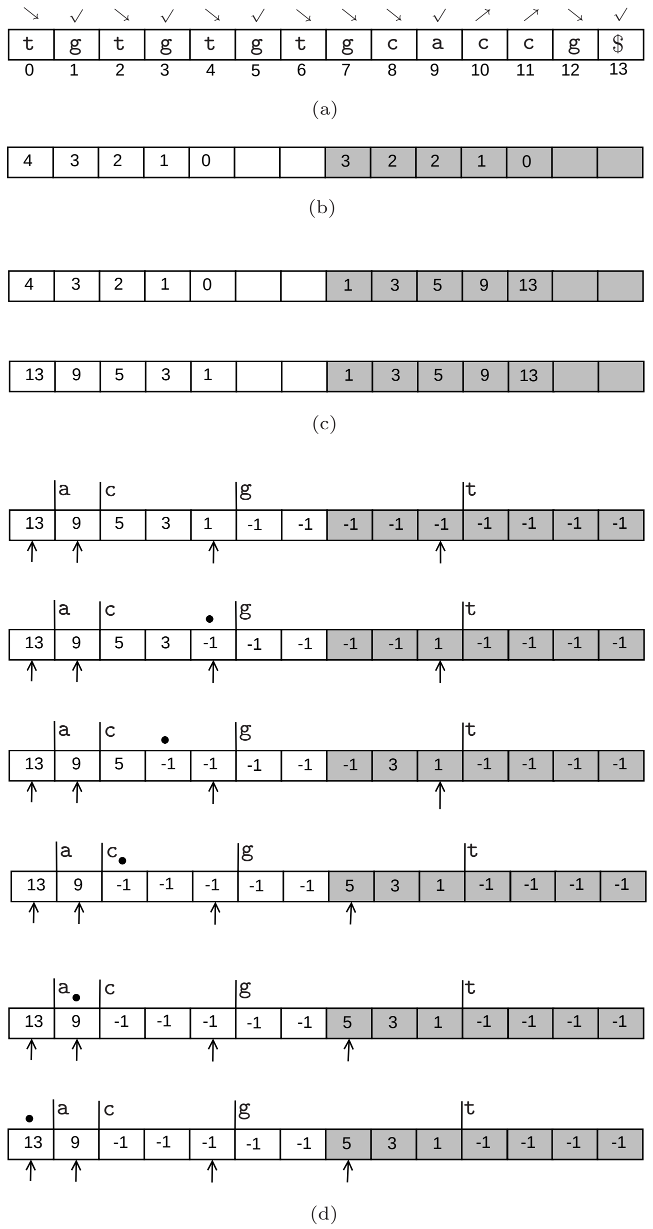

Figure S6: An example of in-place execution of the combine phase. Here the computation has just returned to level 0 from level 1. (a) Text T_0 (b) A_0^l (non-shaded) contains SA^{T_1}, and A_0^r (shaded) contains T_1. We want to compute SA^{T_0} in-place in A_0. (c) The suffixes of T_1 in A_0^l are replaced by their corresponding $\surd$ suffixes in T_0. This is done by overwriting A_0^r with the $\surd$ suffixes of T_0 in the order the appear in T_0; and then for every suffix j in A_0^l, replacing it by the suffix in the j-th position in A_0^r. (d) Scanning A_0^l from right to left and placing the suffix at the scan position (denoted by $\bullet$) into the respective bucket positions indicated by $\uparrow$s in A_0.
